# Daily Sleep and Language Use in Late Life: A Latent Profile Analysis Approach

**DOI:** 10.1093/geroni/igaf122.3094

**Published:** 2025-12-31

**Authors:** Zexi Zhou, Shiyang Zhang, Karen Fingerman

**Affiliations:** The University of Texas at Austin, Austin, Texas, United States; The University of Texas at Austin, Austin, Texas, United States; The University of Texas at Austin, Austin, Texas, United States

## Abstract

Language is crucial for older adults’ cognitive activity and social communication, and everyday sleep may play a role in how they talk. This study examines the associations between cognitive functioning, daily sleep, and language use in late life. We used intensive longitudinal data of 266 older adults over 5–6 days from the Daily Experiences and Well-being Study (Mage = 73.74). At baseline, participants completed a battery of standard cognitive tests. Across the study period, Electronically Activated Recorder recorded participants’ ambient sound 30 seconds every 7 minutes. Every morning, participants reported their sleep the prior night. Linguistic Inquiry and Word Count software was used to generate linguistic features from transcriptions of recorded speech, including word count, complexity (i.e., words per sentence, words more than 6 letters), emotionality (positivity, negativity), cognitive processes, and informal language. Day-level Latent Profile Analysis identified 2 classes of older adults’ daily language: cognitively engaged complex language (i.e., more words, more complex language, higher cognitive processing; 90%) and emotionally expressive informal language (i.e., simpler language, more emotional and informal expressions; 10%). Older adults were more likely to generate cognitively engaged complex language when they had longer-than-usual (versus shorter-than-usual) sleep the prior night, especially for those with better cognitive functioning. Reporting higher-quality prior night’s sleep was also linked to a greater likelihood of generating cognitively engaged complex language regardless of cognitive functioning. Findings suggest specific patterns of daily language use in late life, and highlight the importance of sleep in supporting cognitively engaged language use among older adults.

